# Supplementary material for: Mesoscopic cortical network reorganization during recovery of optic nerve injury in GCaMP6s mice
Source: Sci Rep. 2020 Dec 8;10:21472. doi: 10.1038/s41598-020-78491-z (PMC7723052; doi:10.1038/s41598-020-78491-z)
Supplement: Supplementary file 2 — Supplementary Information. [file 41598_2020_78491_MOESM2_ESM.docx]

**Supplementary material**

**Mesoscopic cortical network reorganization during recovery of optic nerve injury in GCaMP6s mice**

Marianne Groleau^1^, Mojtaba Nazari-Ahangarkolaee^2^, Matthieu P. Vanni^3^, Jacqueline L. Higgins^1^, Anne-Sophie Vézina Bédard^1^, Bernhard A. Sabel^5^, Majid H. Mohajerani^2*^, Elvire Vaucher^1*^

**Legend Figure suppl. Figure 1, suppl. Table 1 and Table 2**

**Suppl. Figure 1** **Amplitude responses in both hemisphere upon stimulation of the left or right eye following optic nerve injury**. Upper panel for cortical peak response (ΔF/F_0_ x 100) upon stimulation of the left eye and lower panel amplitude response upon stimulation of the left eye. **A.** Cortical peak response (ΔF/F_0_ x 100) in the contralateral hemisphere to the flash stimulation in the injured eye; **B.** Cortical peak response (ΔF/F_0_ x 100) in the ipsilateral hemisphere to the flash stimulation in the injured eye **C**. Cortical peak response (ΔF/F_0_ x 100) in the ipsilateral hemisphere to the flash stimulation in the healthy eye; **D.** Cortical peak response (ΔF/F_0_ x 100) in the contralateral hemisphere to the flash stimulation in the healthy eye. V1, primary visual cortex; A, AM, PM, anterior, anteromedial, and posteromedial regions of the secondary visual cortex; AC, anterior cingulate cortex; RS, retrosplenial cortex.

**Suppl. Table 1 Calcium imaging values of the non-visual sensorimotor and motor areas**

| Days from ONC | -2 | | | 1h | | 1 | | 3 | | 5 | | 7 | | 14 | | 23 | | 31 |
| --- | --- | --- | --- | --- | --- | --- | --- | --- | --- | --- | --- | --- | --- | --- | --- | --- | --- | --- |
| **Amplitude** | |  |  | |  | |  | |  | |  | |  | |  | |  | |
| HL **contra.** | 1.58±0.18 | | | 1.37±0.17 | | 0.61±0.10 | | 0.59±0.09 | | 0.70±0.09 | | 0.91±0.14 | | 1.01±0.13 | | 0.56±0.07 | | 0.88±0.08 |
| HL **ipsi.** | 1.40±0.12 | | | 1.29±0.13 | | 0.57±0.08 | | 0.54±0.11 | | 0.45±0.08 | | 0.64±0.08 | | 0.93±0.13 | | 0.33±0.07 | | 0.47±0.06 |
| M1 **contra.** | 0.48±0.05 | | | 0.62±0.06 | | 0.17±0.04 | | 0.18±0.03 | | 0.36±0.06 | | 0.39±0.03 | | 0.43±0.07 | | 0.44±0.06 | | 0.52±0.06 |
| M1 **ipsi.** | 0.41±0.04 | | | 0.43±0.05 | | 0.26±0.04 | | 0.15±0.03 | | 0.47±0.05 | | 0.44±0.02 | | 0.46±0.05 | | 0.36±0.04 | | 0.39±0.05 |
| **Peak latency** | |  |  | |  | |  | |  | |  | |  | |  | |  | |
| HL **contra.** | 0.43±0.02 | | | 0.43±0.03 | | 0.24±0.04 | | 0.20±0.03 | | 0.31±0.04 | | 0.29±0.04 | | 0.27±0.03 | | 0.29±0.04 | | 0.33±0.03 |
| HL **ipsi.** | 0.44±0.02 | | | 0.52±0.03 | | 0.32±0.04 | | 0.13±0.02 | | 0.26±0.04 | | 0.15±0.03 | | 0.24±0.03 | | 0.16±0.03 | | 0.26±0.04 |
| M1 **contra.** | 0.29±0.03 | | | 0.45±0.04 | | 0.22±0.06 | | 0.18±0.03 | | 0.26±0.04 | | 0.33±0.04 | | 0.22±0.03 | | 0.23±0.04 | | 0.22±0.06 |
| M1 **ipsi.** | 0.26±0.03 | | | 0.38±0.04 | | 0.11±0.02 | | 0.17±0.03 | | 0.36±0.03 | | 0.29±0.02 | | 0.29±0.03 | | 0.36±0.04 | | 0.23±0.03 |

Calcium signal (mean±s.e.m.) for the sensorimotor areas during the early recovery after the ONC. **Amplitude** Cortical peak response (ΔF/F_0_x100) following the flash stimulation in the injured eye; **Peak latency** Peak latency (sec) following the flash stimulation in the injured eye; ONC: optic nerve crush; HL **contra.** or **ipsi.,** hindlimb sensory cortex in the contralateral or ipsilateral hemisphere to stimulation; M1 **contra.** or **ipsi.,** primary motor cortex in the contralateral or ipsilateral hemisphere to stimulation.

**Supplemental Table 2:** Raw data of the optomotor task after an unlilateral ONC in the left eye

| Day from ONC | -7 | -4 | 0 | 1 | 3 | 7 | 14 | 21 | 28 |
| --- | --- | --- | --- | --- | --- | --- | --- | --- | --- |
| **Right eye (OD)** |  |  |  |  |  |  |  |  |  |
| Mouse A | 0.48 | 0.46 |  | 0.49 | 0.50 | 0.49 | 0.51 | 0.53 | 0.55 |
| B | 0.44 | 0.39 | 0.47 | 0.51 | 0.48 | 0.51 | 0.48 | 0.49 | 0.49 |
| C | 0.45 | 0.44 |  | 0.48 | 0.50 | 0.51 | 0.52 | 0.53 | 0.53 |
| D | 0.43 | 0.41 |  | 0.46 | 0.49 | 0.50 | 0.51 | 0.48 | 0.56 |
| E | 0.43 | 0.32 | 0.41 | 0.46 | 0.48 | 0.42 | 0.44 | 0.49 | 0.46 |
| F | 0.41 | 0.44 |  | 0.49 | 0.49 | 0.49 | 0.42 | 0.47 | 0.44 |
| G | 0.44 | 0.45 |  | 0.49 | 0.48 |  | 0.44 | 0.34 | 0.39 |
| H | 0.38 | 0.38 |  | 0.46 | 0.46 | 0.48 | 0.35 | 0.45 | 0.47 |
| I | 0.47 | 0.48 |  | 0.51 | 0.51 | 0.50 | 0.51 | 0.54 | 0.54 |
|  |  |  |  |  |  |  |  |  |  |
| **Left eye**  **(OS)** |  |  |  |  |  |  |  |  |  |
| Mouse A | 0.43 | 0.45 |  | 0.00 | 0.00 | 0.00 | 0.00 | 0.00 | 0.00 |
| B | 0.37 | 0.41 | 0.46 | 0.00 | 0.00 | 0.00 | 0.00 | 0.00 | 0.00 |
| C | 0.46 | 0.44 |  | 0.00 | 0.00 | 0.00 | 0.00 | 0.00 | 0.00 |
| D | 0.36 | 0.38 |  | 0.00 | 0.00 | 0.00 | 0.00 | 0.00 | 0.00 |
| E | 0.40 | 0.36 | 0.41 | 0.00 | 0.00 | 0.00 | 0.00 | 0.00 | 0.00 |
| F | 0.38 | 0.42 |  | 0.00 | 0.00 | 0.00 | 0.00 | 0.00 | 0.00 |
| G | 0.47 | 0.47 |  | 0.39 | 0.39 |  | 0.36 | 0.39 | 0.38 |
| H | 0.50 | 0.47 |  | 0.00 | 0.00 | 0.00 | 0.00 | 0.00 | 0.00 |
| I | 0.30 | 0.32 |  | 0.00 | 0.00 | 0.00 | 0.00 | 0.00 | 0.00 |
|  |  |  |  |  |  |  |  |  |  |

Values are cpd (cycle per degree). ONC: optic nerve crush; OD. oculus dexter; OS. oculus sinister.
